# Supplementary material for: Potential effects of endogenous RNA/DNA hybrids on CRISPR-Cas9-mediated homology-directed repair
Source: Mol Ther Nucleic Acids. 2026 Feb 27;37(2):102880. doi: 10.1016/j.omtn.2026.102880 (PMC13022658; doi:10.1016/j.omtn.2026.102880)
Supplement: Document S1. Figure S1 [file mmc1.pdf]

OMTN, Volume *37*

Supplemental information

Potential effects of endogenous RNA/DNA hybrids on CRISPR-Cas9-mediated homology-directed repair

Francesco Puzzo, Batuhan Bayram, Claudia Macaubas, Angela Lin, Hagoon Jang, Feijie Zhang, Elizabeth Mellins, and Mark A. Kay

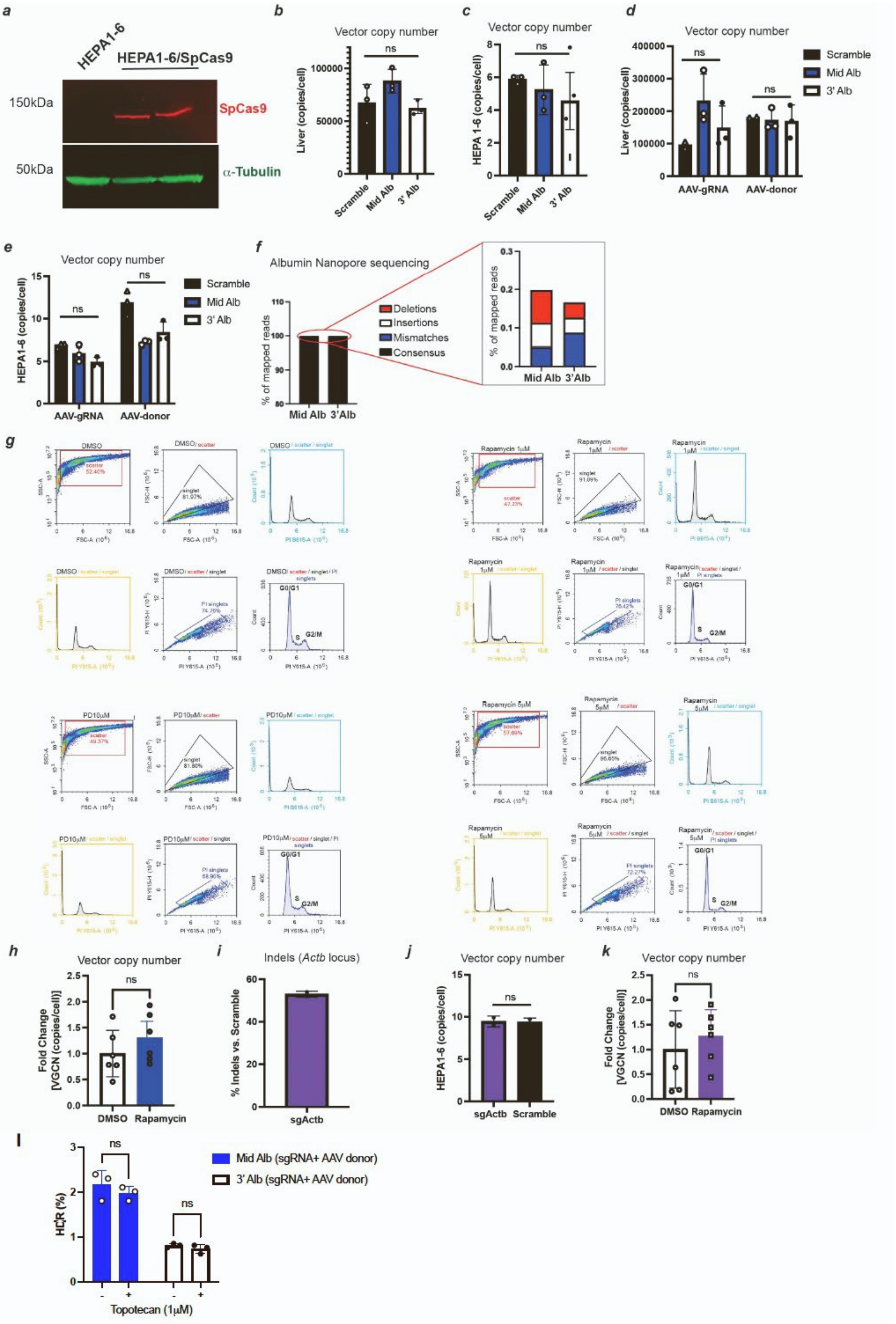
a
HEPA1-6
HEPA1-6/SpCas9
150kDa
SpCas9
50kDa
α-Tubulin
b
Vector copy number
Liver (copies/cell)
ns
Scramble
Mid Alb
3' Alb
c
Vector copy number
HEPA 1-6 (copies/cell)
ns
d
Vector copy number
Liver (copies/cell)
AAV-gRNA
AAV-donor
e
Vector copy number
HEPA1-6 (copies/cell)
f
Albumin Nanopore sequencing
% of mapped reads
Deletions
Insertions
Mismatches
Consensus
Mid Alb
3'Alb
g
DMSO
PD10μM
Rapamycin 1μM
Rapamycin 5μM
scatter
singlet
PI singlets
G0/G1
S
G2/M
h
Vector copy number
Fold Change [VGCN (copies/cell)]
DMSO
Rapamycin
i
Indels (Actb locus)
% Indels vs. Scramble
sgActb
j
Vector copy number
HEPA1-6 (copies/cell)
sgActb
Scramble
k
Vector copy number
Fold Change [VGCN (copies/cell)]
DMSO
Rapamycin
l
HDR (%)
Mid Alb (sgRNA+ AAV donor)
3' Alb (sgRNA+ AAV donor)
Topotecan (1μM)

Figure S1. Analysis of AAV vector copy number and editing outcomes

a. Western blot analysis showing the expression of SpCas9 in HEPA1-6/SpCas9 cells. HEPA1-6 were used as negative control. Quantification of AAV vector copy number (VCN) in mouse liver (**b, d)** and HEPA1-6/SpCas9 cells (**c, e**) following administration of *scAAVDJ-sgMid Alb* or *scAAVDJ-sg3' Alb*, alone (**b, c**) or in combination with *ssAAVDJ-donor Mid Alb* and *ssAAVDJ-donor 3' Alb* (**d, e**). **f**. Nanopore analysis of integrants at the *Albumin* locus in HEPA1-6/SpCas9 cells transduced for 72 h with 10,000 vg/cell of *scAAVDJ-sgMid Alb* + 10,000 vg/cell of *ssAAVDJ-donor Mid Alb*, or 10,000 vg/cell of *scAAVDJ-sg3' Alb* + 10,000 vg/cell of *ssAAVDJ-donor 3' Alb.* **g.** Representative flow cytometry plots showing gating strategy for HEPA1-6 cells for cell cycle analysis using propidium iodide staining (PI). **h.** Fold change in HEPA1-6/SpCas9 cells VCN transduced with *scAAVDJ-sgMid Alb* + *ssAAVDJ-donor Mid Alb*, or *scAAVDJ-sg3' Alb* + *ssAAVDJ-donor 3' Alb*. upon treatement with rapamycin. DMSO-treated cells were used as control. **i.** Indel frequency at the *Actb* locus in HEPA1-6/SpCas9 cells transduced with *scAAVDJ-sgActb* compared to scramble control. **j.** AAV vector copy number in HEPA1-6/SpaCas9 cells transduced with *scAAVDJ-sgActb* or scramble control. **k.** Fold change in HEPA1-6/SpCas9 cells VCN transduced with 10,000vg/cell of s*scAAVDJ-sgActb* + *ssAAVDJ-donor Actb* upon treatment with rapamycin. DMSO-treated cells were used as control. **l.** ddPCR detection of HDR events in HEPA1-6/SpCas9 cells pre-treated with 1 µM topotecan for 1 h, followed by 72 h transduction with 10,000 vg/cell of scAAVDJ-sgMid Alb + 10,000 vg/cell ssAAVDJ-donor Mid Alb, or scAAVDJ-sg3′ Alb + ssAAVDJ-donor 3′ Alb.

Statistical analysis: **b- e.** One-way ANOVA with Dunnett's post hoc test; **h, j, k.** Student's t-test. *p*<0.05, **p*<0.01, ***p*<0.001, ****p*<0.0001, ns=non statistically significant. Error bars represent mean ± SD.

Table S1 (see file excel published along with supplemental material) contains:

- The raw data from the Nanopore sequencing experiment showed in figure S1f
- The sequences of the oligonucleotides used in the present study
