# Supplementary material for: Potential effects of endogenous RNA/DNA hybrids on CRISPR-Cas9-mediated homology-directed repair
Source: Mol Ther Nucleic Acids. 2026 Feb 27;37(2):102880. doi: 10.1016/j.omtn.2026.102880 (PMC13022658; doi:10.1016/j.omtn.2026.102880)
Supplement: Document S2. Article plus supplemental information [file mmc3.pdf]

Molecular Therapy
Nucleic Acids
Brief report

Potential effects of endogenous RNA/DNA hybrids on CRISPR-Cas9-mediated homology-directed repair

Francesco Puzzo,[1,2] Batuhan Bayram,[2] Claudia Macaubas,[2] Angela Lin,[1,2] Hagoon Jang,[1,2] Feijie Zhang,[1,2] Elizabeth Mellins,[2,3] and Mark A. Kay[1,2]

[1]Department of Genetics, Stanford University, Stanford, CA 94305, USA; [2]Department of Pediatrics, Stanford University, Stanford, CA 94305, USA

Recombinant adeno-associated virus (rAAV) vectors and CRISPR-Cas9 are widely used in gene therapy. However, how endogenous DNA secondary structures may potentially affect genome editing outcomes is not fully understood. RNA/DNA hybrids (R-loops), which form mostly during transcription, are dynamically regulated in cells and have been implicated in influencing DNA repair pathway choice. Here, we investigated whether genomic R-loops are associated with differences in Cas9-mediated genome editing outcomes *in vitro* and *in vivo*. By targeting regions with relatively low or high R-loop levels within the murine albumin (*Alb*) and actin (*Actb*) loci, we observed comparable insertion/deletion (indel) frequencies across sites with different R-loop abundance. In contrast, homology-directed repair (HDR) efficiency appeared reduced at R-loop-enriched sites in proliferating hepatocyte-derived cells (HEPA1-6) but not in quiescent hepatocytes *in vivo*. Manipulations resulting in reducing R-loop levels, including RNaseH1 overexpression or pharmacological induction of G1 arrest were associated with increased HDR at these genomic sites. In addition, T cell activation correlated with elevated R-loop accumulation suggesting they might influence *ex vivo* genome editing. Together, these observations suggest that endogenous R-loop levels might influence HDR efficiency during Cas9-mediated editing and is a parameter to consider when designing genome editing strategies.

INTRODUCTION

Recombinant adeno-associated virus (rAAV) vectors are widely used in gene therapy due to their favorable safety profile and capacity for targeted genomic integration when used in combination with nucleases.[1] However, the integration landscape of rAAV vectors is influenced by local DNA architecture and transcriptional activity,[2] with emerging evidence implicating RNA/DNA hybrid structures, or R-loops, as critical determinants of vector targeting.[3] R-loops form co-transcriptionally when nascent RNA hybridizes to the template DNA strand, and they are increasingly recognized as regulators of genome stability, transcription, and DNA repair.[4,5]

CRISPR-Cas9 is a versatile genome editing platform whose efficiency is similarly influenced by chromatin context, transcriptional activity, and DNA secondary structures.[6] While R-loops-like structures formed by the annealing of the guide RNA (gRNA) and target DNA have been shown to both promote[7] and inhibit[8] Cas9 activity, the impact of endogenous genomic R-loops on Cas9-mediated editing remains poorly understood. Moreover, during transcription, nascent RNA has been associated with homologous recombination (HR),[9,10] and R-loops have been implicated in the regulation of HR,[11,12] suggesting they may also influence homology-directed repair (HDR) outcomes during genome editing.

Here, by targeting loci with low or high R-loop levels, we observed comparable insertions/deletions (indel) frequencies but selectively reduced HDR at high R-loop sites, which could be rescued by RNaseH1-mediated R-loop degradation or by manipulating cell-cycle quiescence. Studies in activated CD4$^+$ T cells further indicated that R-loop dynamics may be relevant in *ex vivo* gene therapy contexts.

Taken together, these results delineate an unanticipated mechanistic interplay between R-loop levels, cell-cycle dynamics, and the efficiency of HDR, establishing R-loops as a potential barrier to optimal CRISPR-Cas9-mediated genome editing.

RESULTS

We previously demonstrated that R-loops strongly influence the integration profile of AAV vectors both *in vitro* and *in vivo* at the *albumin* (*Alb*) locus.[3] In addition, we observed that genomic R-loop levels are markedly higher and more abundant in murine hepatic HEPA1-6 cells compared to mouse liver tissue[3] (Figure 1A). To assess whether R-loops also affect the outcome of CRISPR-Cas9-mediated genome editing, we employed HEPA1-6 cells (HEPA1-6/SpCas9) (Figure S1A) and transgenic mice constitutively expressing

Received 9 October 2025; accepted 24 February 2026;
https://doi.org/10.1016/j.omtn.2026.102880.

[3]Deceased

Correspondence: Mark A. Kay, Department of Genetics, Stanford University, Stanford, CA 94305, USA.
E-mail: markay@stanford.edu

© 2026 The Authors.
Published by Elsevier Inc. on behalf of The American Society of Gene and Cell Therapy.
This is an open access article under the CC BY license (http://creativecommons.org/licenses/by/4.0/).

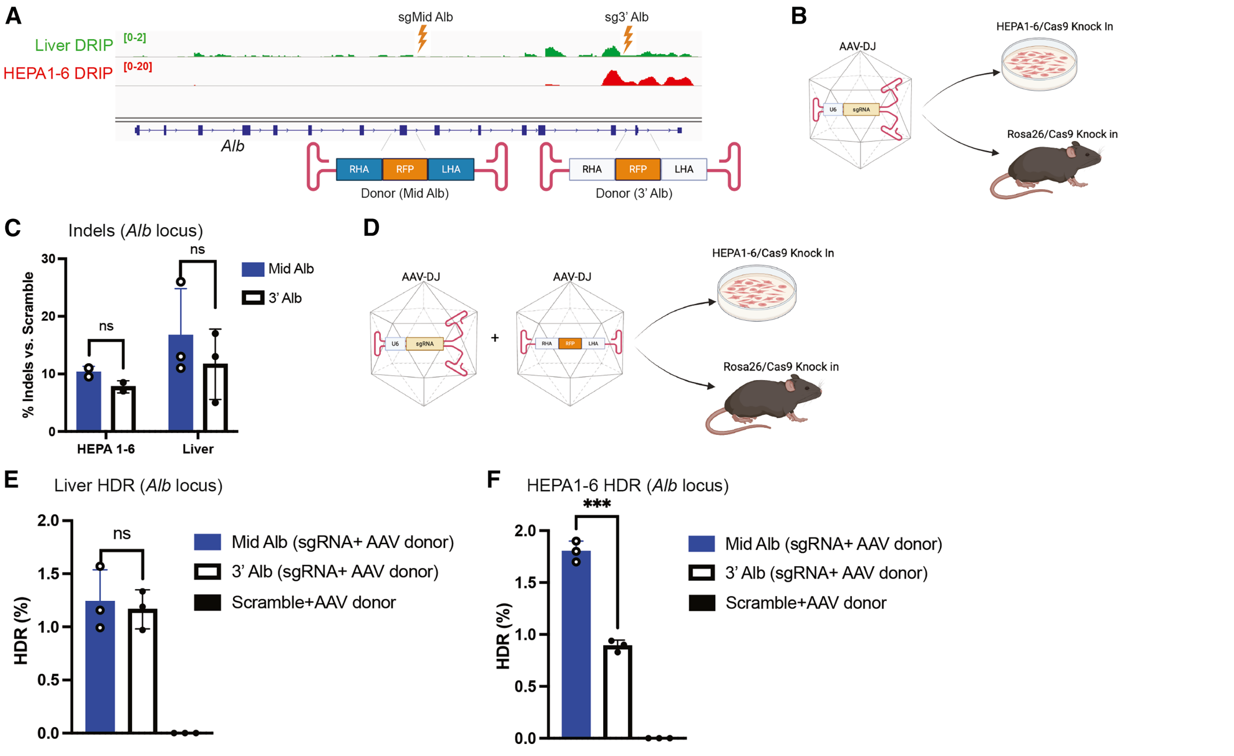

Figure 1. Targeting the *Alb* locus with AAV-delivered CRISPR-Cas9 and donor DNA

(A) IGV visualization of the DRIP signal at the *Alb* locus and schematic of donor DNA constructs targeting mid (donor Mid Alb) and 3′ (donor 3′ Alb) regions. Liver (0–2) and HEPA1-6 (0–20) samples are shown using distinct axis scaling to accurately represent and highlight the different levels of R-loops between the two conditions. (B) Schematic overview of *in vitro* and *in vivo* experimental strategies for detecting indels following delivery of self-complementary AAVDJ-sgRNAs (scAAVDJ-sgMid Alb and scAAVDJ-sg3′ Alb). (C) Indel analysis in HEPA1-6/SpCas9 cells transduced for 72 h with 10,000 vg/cell of scAAVDJ-sgMid Alb or scAAVDJ-sg3′ Alb, and in livers of Cas9 transgenic mice treated for 8 days with 1×10^{11} vg/mouse of the same vectors. (D) Experimental design for assessing HDR following co-delivery of scAAVDJ-sgRNAs and single-stranded AAVDJ donor templates (ssAAVDJ-donor Mid Alb and ssAAVDJ-donor 3′ Alb*)*. (E) Detection of HDR events by ddPCR in livers of Cas9 transgenic mice treated for 15 days with 1×10^{11} vg/mouse scAAVDJ-sgMid Alb + 1×10^{11} vg/mouse ssAAVDJ-donor Mid Alb, or with 1×10^{11} vg/mouse scAAVDJ-sg3′ Alb + 1×10^{11} vg/mouse ssAAVDJ-donor 3′ Alb. A scrambled gRNA vector with donor served as negative control. (F) Detection of HDR events by ddPCR in HEPA1-6/SpCas9 cells transduced for 72 h with 10,000 vg/cell of scAAVDJ-sgMid Alb + 10,000 vg/cell of ssAAVDJ-donor Mid Alb, or 10,000 vg/cell of scAAVDJ-sg3′ Alb + 10,000 vg/cell of ssAAVDJ-donor 3′ Alb. Scrambled gRNA with donor served as negative control. Statistical analysis: (C) two-way ANOVA with Sidak's post hoc test; (E and F) Student's *t* test. $p < 0.05$, $*p < 0.01$, $**p < 0.001$, $***p < 0.0001$. Error bars represent mean ± SD.

Streptococcus pyogenes Cas9 (SpCas9).[13] Given the critical role of R-loops in both promoting[7] and inhibiting[8] CRISPR-Cas9 enzymatic activity, we first sought to determine whether endogenous genomic R-loops could affect SpCas9 function. To investigate the potential influence of R-loop abundance on CRISPR-Cas9 editing efficiency, we designed two gRNAs targeting distinct regions of the *Alb* locus. One was directed to the central region (sgMid-Alb; intron 8), which exhibits low R-loop formation, and another directed to the 3′ region (sg3′-Alb; exon 13), where R-loops were enriched (Figure 1A). Self-complementary AAV-DJ (scAAV-DJ) vectors[14] were then constructed to deliver these gRNAs into HEPA1-6/SpCas9, as well as into Cas9 mouse liver *in vivo* (Figure 1B; Figures S1B and S1C). Analysis of genome editing outcomes revealed comparable indel frequencies (10%–15%) across both experimental systems, and no significant difference in editing efficiency was observed between the mid and 3′ regions of *Alb*, despite their differences in R-loop abundance (Figure 1C).

Recent studies have highlighted a critical role for RNA in the regulation of HR.[9,10] Based on this, we investigated whether R-loops might influence CRISPR-Cas9-mediated HDR during genome editing. To this end, we utilized the two previously designed gRNAs in combination with two DNA donor templates targeting either the mid or 3′ region of the *Alb* locus (Figure 1A). The donor DNAs were packaged into single-stranded AAV-DJ (ssAAV-DJ) vectors and co-delivered alongside the scAAVs expressing the gRNAs into both HEPA1-6/SpCas9 cells and Cas9 mice (Figure 1D; Figures S1D and S1E). Molecular assessment by digital droplet PCR (ddPCR) of HDR events revealed no significant difference in integration efficiency between the mid and 3′ *Alb* loci *in vivo* (Figure 1E). However, in HEPA1-6/SpCas9 cells, HDR efficiency was significantly higher at the mid *Alb* region compared to the 3′ *Alb* region (Figure 1F). This reduced HDR efficiency at the 3′ *Alb* site may be potentially attributed to the remarkably elevated levels of R-loops observed at this locus especially at the 3′ end in

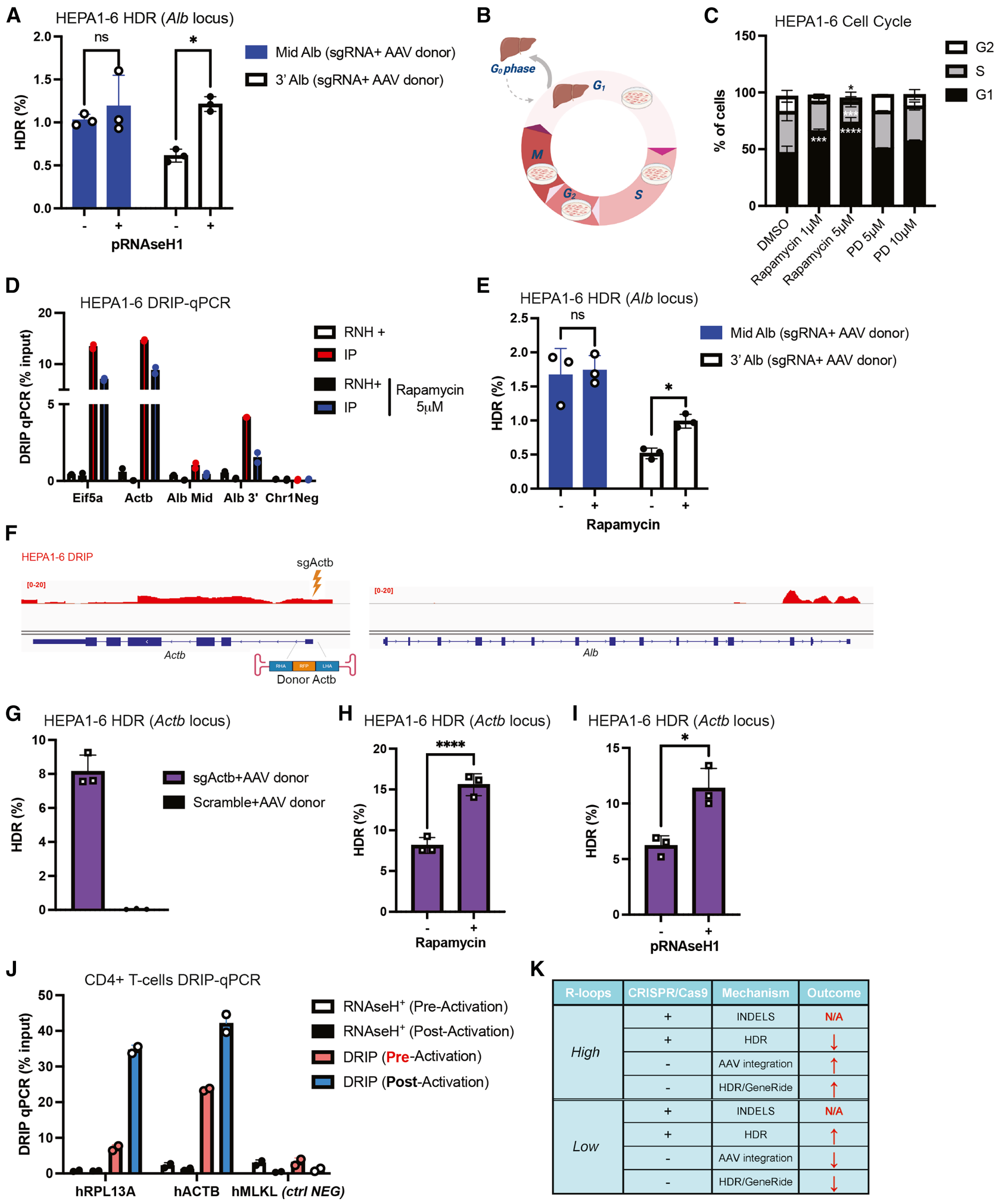

Figure 2. Modulation of HDR efficiency by R-loop resolution, cell-cycle perturbation, and locus context

(A) ddPCR quantification of HDR events in HEPA1-6/SpCas9 cells transiently overexpressing human RNase H1 and transduced with 10,000 vg/cell of scAAVDJ-sgMid Alb + 10,000 vg/cell ssAAVDJ-donor Mid Alb, or 10,000 vg/cell of scAAVDJ-sg3′ Alb + 10,000 vg/cell ssAAVDJ-donor 3′ Alb. (B) Schematic representation of cell cycle profiles in HEPA1-6 cells and murine hepatocytes. (C) Flow cytometry analysis of HEPA1-6/SpCas9 cells treated for 24 h with rapamycin (1 μM, 5 μM) or palbociclib (PD; 5 μM, 10 μM). DMSO-treated cells served as controls. (D) DRIP-qPCR analysis of HEPA1-6/SpCas9 cells after 24 h treatment with 5 μM rapamycin; DMSO-treated cells served as controls. (E) ddPCR detection of HDR events in HEPA1-6/SpCas9 cells pre-treated with 5 μM rapamycin for 24 h, followed by 72 h transduction with 10,000 vg/cell of scAAVDJ-sgMid Alb + 10,000 vg/cell ssAAVDJ-donor Mid Alb, or scAAVDJ-sg3′ Alb + ssAAVDJ-donor 3′ Alb. (F) IGV visualization of the *Alb* and *Actb* loci showing gRNA (sgActb) and donor (donor Actb) design targeting the 5′ region of *Actb*. (G) ddPCR detection of HDR events in HEPA1-6/SpCas9 cells transduced for 72 h with 10,000 vg/cell of scAAVDJ-sgActb + 10,000 vg/cell ssAAVDJ-donor Actb. Scrambled gRNA with donor served as negative control. (H) ddPCR detection of HDR events in HEPA1-6/SpCas9 cells pre-treated with 5 μM rapamycin for 24 h, followed by 72 h transduction with scAAVDJ-sgActb + ssAAVDJ-donor Actb. (I) ddPCR detection of HDR events in HEPA1-6/SpCas9

(legend continued on next page)

HEPA1-6/SpCas9 cells relative to murine liver tissue *in vivo*[3] (Figure 1A). We next examined whether elevated R-loop accumulation affects the DNA repair mechanisms engaged during HR-mediated genome editing. To assess this, targeted deep sequencing was performed on the mid and 3′ regions of the *Alb* locus following genome editing. This analysis revealed no detectable changes in DNA repair outcomes, as 99.8% of the recovered sequences corresponded to the expected HDR product, while the remaining 0.2% consisted of insertions/deletions (indels) and mismatches (Figure S1F). These data suggest that only a minor fraction of the editing events was resolved through non-homologous end joining (NHEJ). However, the possible contribution of microhomology-mediated end joining (MMEJ) cannot be ruled out, as integration through microhomology can result in deletion events. Furthermore, the absence of detectable integrated inverted terminal repeat (ITR) sequences in this analysis is most likely attributable to the limited sequencing depth of the nanopore dataset, which comprised approximately 6,000 reads.

Next, to investigate whether R-loop levels could modulate CRISPR-Cas9-mediated HDR, we transiently overexpressed RNaseH1 in HEPA1-6/SpCas9 cells via plasmid transfection[3] and quantified HDR frequencies at both the mid and 3′ regions of the *Alb* locus. RNaseH1 enzymatically degrades RNA/DNA hybrids, thereby globally reducing R-loop levels.[15] Notably, RNaseH1 overexpression had no measurable impact on HDR efficiency at the mid *Alb* region but significantly increased HDR outcomes at the 3′ *Alb* site (Figure 2A).

These results led us to hypothesize that the elevated R-loop levels observed in HEPA1-6 cells, compared with the liver, may be attributable to differences in cell-cycle state, since hepatocytes *in vivo* are predominantly quiescent in G0/G1, whereas immortalized HEPA1-6 cells continuously proliferate and undergo mitosis[16,17] (Figure 2B). To test this hypothesis, we pharmacologically induced quiescence in HEPA1-6/SpCas9 cells using two reported cell-cycle inhibitors, rapamycin[18] and palbociclib (PD).[19] Flow cytometry analysis of propidium iodide-stained cells (Figure S1G) revealed that regardless the dose tested, PD treatment did not arrest cells in G1, whereas rapamycin induced a dose-response accumulation of cells in G1 relative to DMSO-treated controls (Figure 2C). Although palbociclib has been reported as a potent cell-cycle inhibitor,[19] in our experimental setting, the murine origin of the cells and the relatively short treatment duration (24 h) may have limited its efficacy.

We next assessed whether rapamycin modulates R-loop accumulation across transcriptionally active regions. DNA/RNA immunoprecipitation (DRIP)-qPCR analysis demonstrated that treatment with 5 μM rapamycin resulted in a pronounced reduction of R-loop levels, concomitant with the induction of G1 cell-cycle arrest. The decrease in R-loops was particularly evident at highly transcribed loci, including the regions which corresponds to the same genomic sites tested in our HDR experiments (Figure 2D). These findings indicate that rapamycin treatment diminishes R-loop formation at active chromatin regions under conditions that also restrict cell-cycle progression. Similar to RNaseH1 overexpression, rapamycin-mediated R-loop reduction had no effect on HDR at the mid *Alb* locus but significantly enhanced HDR efficiency at the 3′ *Alb* region (Figure 2E). Importantly, rapamycin treatment did not alter the amount of AAV genomes per cell (Figure S1H).

To further validate these findings, we analyzed the *Actb* locus, which displays comparably high R-loop accumulation to the 3′ *Alb* region (Figure 2F). We first confirmed the previously reported sgRNA activity at the 5′ *Actb* site,[20] which yielded ∼50% indels in HEPA1-6/SpCas9 cells (Figure S1I). Following AAV donor transduction (Figure S1J), HDR was observed at ∼8% efficiency at this locus (Figure 2G). Consistent with results at 3′ *Alb*, RNaseH1 overexpression (Figure 2H) and rapamycin treatment significantly increased HDR at the 5′ *Actb* site, and rapamycin treatment (Figure 2I; Figure S1K) enhanced HDR efficiency.

Furthermore, we conducted an experiment using topotecan, a compound known to enhance AAV-mediated genome integration, which concurrently induces a significant increase in R-loop formation at the *Alb* locus.[3] However, no statistically significant differences in HDR efficiency were observed at either the mid *Alb* or 3′ *Alb* regions (Figure S1L).

In *ex vivo* gene therapy, target cells, such as T cells for CAR-T therapies or hematopoietic stem and progenitor cells (HSPCs) for cell-based therapies, must undergo activation to induce proliferation and facilitate efficient transduction with gene therapy vectors or delivery of gene editing complexes, such as Cas9/ribonucleoproteins.[21,22] To investigate R-loop dynamics in a more therapeutically relevant context, we quantified RNA/DNA hybrid formation in $CD4^+$ T cells before and after *ex vivo* activation. Using DRIP-qPCR, we observed a significant increase in R-loop levels at highly transcribed loci upon T cell activation (Figure 2J). While these results may serve as a surrogate for genome-wide R-loop accumulation, further studies are warranted to determine whether the activation-induced increase in R-loops levels could impact the efficiency or fidelity of CRISPR-Cas9-mediated genome editing in *ex vivo* cell therapy settings.

DISCUSSION

Our research showed an unappreciated layer of complexity in CRISPR-Cas9-mediated genome editing, emerging from the impact

cells transiently overexpressing human RNase H1 and transduced with scAAVDJ-sgActb + ssAAVDJ-donor Actb. (J) DRIP-qPCR analysis of $CD4^+$ T cells from a healthy donor, pre- and post-activation with 10 μg/mL PHA, 50 IU/mL IL-2, 5 ng/mL IL-7, and 5 ng/mL IL-15. DRIP, immunoprecipitated samples treated with S9.6 antibody which are enriched in R loops; $RNAseH^+$, samples treated with RNAseH1+S9.9 antibody which are depleted of R-loops. (K) Table summarizing the effects of R-loops levels on indels, HDR, and AAV integration. N/A, not affected; ↑ increase; ↓ = decrease. Statistical analysis: (A and E) multiple *t* test; (C) two-way ANOVA with Dunnett's post hoc test; (G–I) Student's *t* test. $p < 0.05$, $^*p < 0.01$, $^{**}p < 0.001$, $^{***}p < 0.0001$. Error bars represent mean ± SD.

of endogenous R-loops, structures that are both abundant and dynamically regulated in actively proliferating cells.[4] Our prior work established that R-loops affect the integration profile of recombinant AAV vectors.[3] In this study, our findings extended their impact to HDR outcomes during genome editing. Specifically, Cas9-mediated HDR was markedly suppressed at genomic loci enriched in R-loops, such as the 3′ region of *Alb* and 5′ *Actb*. Moreover, we observed that cell-cycle status modulates R-loop abundance, which in turn impacts HDR efficiency. Notably, the effects of R-loops were not uniform but highly locus and context dependent, with discrepancies observed between immortalized hepatocyte-derived cell lines, such as the HEPA1-6, and quiescent hepatocytes *in vivo*.

Although AAV integration, with[3] or without[23] homology arms, was promoted by the presence of R-loops, a key observation of this study is that high levels of R-loops, while dispensable for indel formation, can significantly inhibit nuclease-mediated HDR efficiency at specific genomic loci in liver cells (Figure 2K). Remarkably, this inhibitory effect was abrogated either by overexpression of RNaseH1 or by pharmacological reduction of R-loops through rapamycin-induced G1 arrest in those cells. These observations suggest that R-loops may modulate the DNA repair pathway choice based on the cell transcriptional activity, cell-cycle regulation and DNA-damage response. These phenomena appear to be modulated by R-loops in a locus-dependent manner. We propose that a "buffer zone" of R-loop accumulation exists, beyond which HDR efficiency might be inhibited. Importantly, the strong context dependence of this effect does not contradict the established model in which Cas9-mediated HDR is enhanced in proliferating cells.[24] Instead, our findings suggest that excessive R-loop formation can act as an additional, locus-specific, possibly cell-specific, limiting factor superimposed on the cellular proliferation state. Mechanistically, excessive R-loops could potentially impair HDR by obstructing DNA end resection or limiting the accessibility of donor templates at Cas9-induced double-strand breaks. Conversely, the involvement of R-loops in AAV genomic integration[3,23] may be influenced by the vector's ITRs, which are known to exhibit recombinogenic properties.[25] The unique secondary DNA structures formed by the ITRs could potentially interact with RNA/DNA hybrid structures along the genome, possibly contributing to the integration process.

Despite we have previously observed that the genomic distribution of R-loops was similarly conserved between HEPA1-6 cells and murine hepatocytes,[3] a significant difference was detected in their overall R-loop abundance. Specifically, the immortalized HEPA1-6 cell line exhibited substantially higher R-loop levels compared to liver tissue.[3] While quiescent hepatocytes *in vivo* exhibited relatively sparse R-loop accumulation, and HDR occurred with comparable efficiency across both low- (mid *Alb*) and high-R-loop (3′*Alb*) regions at the *Alb* locus, proliferating HEPA1-6 cells displayed larger R-loop buildup, consisting of selective suppression of HDR at R-loop-rich loci (3′ *Alb* and 5′ *Actb* regions). This dichotomy highlights a plausible novel and understudied role of cell-cycle status in influencing R-loop biology.[26] Non-dividing hepatocytes might possess intrinsic molecular control mechanisms, such as efficient RNA processing, R-loop-resolving helicases, and DNA/RNA hybrid-specific ribonucleases, that limit aberrant R-loop accumulation. In contrast, proliferative cells such as HEPA1-6 may exhibit diminished or defective activity of these pathways, leading to excessive R-loop buildup that can influence DNA repair processes.[5] This cell-cycle-dependent modulation of R-loop abundance could have broader implications for genome stability, as elevated R-loops in proliferating cells are associated with replication stress, DNA damage, and locus-specific modulation of HDR. Importantly, these findings suggest that R-loop dynamics are not static, but rather are intricately tuned to cellular proliferation status, highlighting the possibility of considering both cell-cycle- and R-loop-related proteins in genome regulation *in vivo*.

Ex vivo genome editing strategies, including CAR-T cells and HSPC, require cellular activation and proliferation to achieve robust transduction and editing.[21,22] Our observation that T cell activation increased R-loop levels suggest that current protocols may need to consider that proliferative states, which facilitate viral vector and genome editing complexes delivery, could possibly impair HDR efficiency at specific genomic loci through significant R-loop accumulation. Furthermore, the excessive cell proliferation along with the delivery of viral vectors and genome editing complexes into *ex vivo* HSPCs have been shown to be detrimental for the cells engraftment in pre-clinical settings.[27] Although further mechanistic studies are warranted, these findings support the exploration of transient R-loop modulation as a strategy to potentially enhance HDR-dependent genome editing. If these findings are generalizable to primary cells, even modest gains in HDR efficiency could have a meaningful impact on therapeutic efficacy and possibly allow for a reduction in the dose required for gene editing systems and vector delivery.

Although restricted to a specific cell type and species, the present work potentially redefines the role of R-loops as active determinants of genome editing outcomes. The paradoxical duality of R-loops, facilitating recombination in some contexts[11] while inhibiting HDR in others,[12] underscores their role as context-specific modulators of DNA repair.[5] R-loops might therefore be considered an additional layer of regulation by integrating cellular state, transcriptional activity, and DNA-repair pathways to influence genome editing outcomes.

Finally, our findings raise important mechanistic questions. Do R-loops directly impair HDR initiation, or do they instead bias DNA repair toward alternative pathways? Moreover, the influence of R-loops may be extended to other emerging CRISPR platforms, including prime and base editors, which also depend on DNA repair and replication machinery.[28] Addressing these questions will be important for a better and general mechanistic understanding, and the rational optimization of genome editing technologies.

In summary, we revealed that R-loops are potential regulators of CRISPR-Cas9-mediated HDR, exerting context-dependent effects that may influence both experimental outcomes and therapeutic

efficacy. By bridging R-loop metabolism and cell biology with genome engineering, our study highlighted the necessity of considering RNA/DNA hybrid dynamics when designing editing strategies, especially in proliferative *ex vivo* clinical settings. These findings open new directions for improving the safety and efficacy of genome editing through potential modulation of R-loop homeostasis.

MATERIALS AND METHODS

Plasmids construction

gRNA expression plasmids were generated using scAAV-sgRNA-GFP[14] (Addgene plasmid #177935). Annealed oligonucleotides[29] were cloned into the BpiI restriction site to produce constructs targeting the *Alb* (mid and 3′ regions) and *Actb* loci.

Donor DNA plasmids were assembled via Gibson cloning using NEBuilder HiFi DNA Assembly Master Mix, with homology arms derived from previously described AAV-HR[3] and Actb donor templates.[20] Each donor contained an RFP expression cassette driven by the INS84 promoter.[3] For transient transfection studies, ppy-CAG_RNaseH1_WT plasmid (Addgene #111906) was utilized.[15]

AAV vector production

AAV vectors were produced in Expi293 suspension cells (Thermo Fisher Scientific, A14527) transfected at 1.5×10^6 cells/mL in 100 mL using 30 μL Rep/Cap, 60 μL pAd5, and 30 μL pAAV plasmids complexed with FectoVIR (VWR, 76469-810) transfection reagent. 72 h post-transfection, AAV were purified using AAVpro Purification Kit (Takara, 6666). Vector genome titers and copy numbers were determined by qPCR using Brilliant III Ultra-Fast SYBR (Agilent Technologies, 600882).

HDR events detection and indels analyses

DNA was extracted using DNeasy Blood & Tissue Kit (Qiagen, 69504). Indels frequencies were quantified using the ICE online tool (https://ice.editco.bio/#/). HDR events were analyzed by droplet digital PCR (ddPCR; Bio-Rad)[30] following HindIII (Thermo Fisher Scientific, FD0505) digestion of 1 μg genomic DNA. Reactions contained FAM- and HEX-labeled probes specific for donor and endogenous *Alb* alleles, respectively, and the HDR frequency was calculated as the HEX/FAM ratio. The ddPCR cycling program was optimized to amplify a 700 bp amplicon for the *Alb* locus: step 1—95°C for 10 min, ramp 2°C/s, step 2—94°C for 30 s, ramp 2°C/s, step 3—60°C for 30 s, ramp 2°C/s, step 4—72°C for 3 min, ramp 2°C/s, step 5—repeat steps 2–4 for 45 cycles, step 6—98°C for 10 min, step 7—4°C. Given the GC-rich region in the amplicon of *Actb* the step 2 of the described program for the *Alb* was modified as follow: step 2—94°C for 60 s, ramp 2°C/s.

Long-read sequencing of *Alb* integrants was performed using the long-read sequencing technology from Oxford Nanopore Technologies (ONT) by

- constructing an amplification-free long-read sequencing library
- sequencing the library with a primer-free protocol using the most accurate R10.4.1 flow cells
- achieving a depth of 6,000 reads per sample (https://plasmidsaurus.com/premium_PCR_sequencing).

Raw signal data were base-called using ONT high-accuracy base calling model, and resulting reads were aligned to a custom reference containing the Mid-*Alb* and 3′-*Alb* target loci.

Processed sequences were aligned to the reference with minimap2 (v.2.30-r1287; -ax map-ont). SAM files were converted to BAM, sorted and indexed, and pileup files were generated with SAMtools (v.1.22.1). Per-position counts of matches, mismatches, insertions, and deletions were computed from the pileup base strings by counting "./," "A/C/G/T/N" (case insensitive), "+," and "-," respectively. Percentage of indels and mismatches were calculated by averaging the number of deletions, insertions, and mismatches and dividing them by the total number of reads per base.

DRIP

DRIP assays were performed as previously described.[3] Immunoprecipitated RNA/DNA was subjected to qPCR for region-specific R-loops quantification. Signal visualization was performed using IGV_2.8.6.

Cell-cycle experiments

HEPA1-6/SpCas9 cells were treated with rapamycin (Selleck, S1039) or palbociclib (Selleck, S1116) for 24 h, fixed in 70% ethanol, stained with propidium iodide, and analyzed by flow cytometry (NovoCyte Penteon, Agilent). Data were processed using NovoExpress software.

In vivo studies

Animal experiments were approved by the Stanford University Administrative Panel on Laboratory Animal Care. Male Rosa26-Cas9 knock-in mice (JAX, 024858) (6–8 weeks old) received 1×10^{11} vg/mouse of AAV intravenously. Animals were euthanized 8 days post-injection for indel analysis or 15 days for HDR assessment. Livers were perfused with PBS, snap-frozen, and homogenized for downstream molecular analyses using bullet blender (Next Advance; Liver: 5′ at speed 10) at 4°C.

In vitro studies

Stable Cas9-expressing HEPA1-6 cells were generated by lentiviral transduction, followed by selection with 15 μg/mL blasticidin. Cells were maintained in DMEM supplemented with 10% fetal bovine serum (FBS), L-glutamine, and antibiotics. AAV transductions were performed at MOI of 10,000 vg/cell. For immunoblotting, cell lysates were prepared in RIPA buffer and probed with antibodies against SpCas9 (AbCam, ab191468) and tubulin (Sigma, T9026). Transient RNaseH1 overexpression was achieved by plasmid transfection 24 h prior AAV exposure.[3] For HDR enhancement experiments, rapamycin or palbociclib diluted in DMSO were applied 24 h before transduction. Topotecan diluted in DMSO was applied on cells for 1 h prior AAV transduction.

T cell experiment

Whole blood was obtained from one healthy donor and collected at the Stanford Blood Center.

Cells were isolated from the Buffy Coats sample by negative selection, using the $CD4^+$ cell isolation kit, according to the manufacturer's instructions (Miltenyi Biotec, 130-096-533). Isolated $CD4^+$ T cells were resuspended at 1×10^6 cells/mL in warm complete RPMI medium (Thermo Fisher, A1049101) supplemented with 10% FBS. 100 μL (100.000 cells) were added to each well of a 96 U bottom TC plate and incubated for 24 h in an incubator at 37°C with 5% CO_2. The day after, 3×10^6 cells were harvested and frozen. The rest of the cells were activated by adding 10 μg/mL of PHA (Millipore Sigma, L1668), 50 IU/mL of IL-2 (BioLegend, 589102), 5 ng/mL of IL-7 (BioLegend, 581902), and 5 ng/mL of IL-15 (BioLegend, 570302). 72 h after, when the number of the cells was doubled, the T cells were harvested and the DNA extraction for the DRIP protocol was started.

Statistics

Statistical analyses were performed using GraphPad Prism. Data were analyzed using parametric one-way or two-way ANOVA with Tukey's or Sidak's post hoc correction ($\alpha = 0.05$). For comparisons between two groups, unpaired *t* tests were used. Data are presented as mean ± standard deviation.

DATA AND CODE AVAILABILITY

All data generated for this study are included in this published manuscript and relative supplemental information.

ACKNOWLEDGMENTS

We would like to thank the Stanford Genomics Facility for the digital droplet PCR instruments and the Stanford FACS facility for the use of the Penteon flow cytometer. This work was supported by grants from the National Institutes of Health, R01-HL064272 to M.A.K.

AUTHOR CONTRIBUTIONS

F.P. and M.A.K. conceived, directed the study, and contributed to the interpretation of results and the significance of the work. F.P. performed all the experiments, analyzed the data, and helped F.Z. who did the *in vivo* work. A.L. analyzed the nanopore sequencing data. H.J. provided the HEPA1-6 constitutively expressing SpCas9. E.M., C.M., and B.B. shared reagents and isolated the T cells. F.P. wrote the manuscript.

DECLARATION OF INTERESTS

The authors do not have any competing interest to disclose.

SUPPLEMENTAL INFORMATION

Supplemental information can be found online at https://doi.org/10.1016/j.omtn.2026.102880.

REFERENCES

1. Wang, D., Tai, P.W.L., and Gao, G. (2019). Adeno-associated virus vector as a platform for gene therapy delivery. Nat. Rev. Drug Discov. *18*, 358–378. https://doi.org/10.1038/S41573-019-0012-9.

2. Nakai, H., Montini, E., Fuess, S., Storm, T.A., Grompe, M., and Kay, M.A. (2003). AAV serotype 2 vectors preferentially integrate into active genes in mice. Nat. Genet. *34*, 297–302. https://doi.org/10.1038/ng1179.

3. Puzzo, F., Crossley, M.P., Goswami, A., Zhang, F., Pekrun, K., Garzon, J.L., Cimprich, K.A., and Kay, M.A. (2024). AAV-mediated genome editing is influenced by the formation of R-loops. Mol. Ther. *32*, 4256–4271. https://doi.org/10.1016/j.ymthe.2024.09.035.

4. Brickner, J.R., Garzon, J.L., and Cimprich, K.A. (2022). Walking a tightrope: The complex balancing act of R-loops in genome stability. Mol. Cell *82*, 2267–2297. https://doi.org/10.1016/j.molcel.2022.04.014.

5. Wulfridge, P., and Sarma, K. (2024). Intertwining roles of R-loops and G-quadruplexes in DNA repair, transcription, and genome organization. Nat. Cell Biol. *26*, 1025–1036. https://doi.org/10.1038/s41556-024-01437-4.

6. Wang, J.Y., and Doudna, J.A. (2023). CRISPR technology: A decade of genome editing is only the beginning. Science *379*, eadd8643. https://doi.org/10.1126/science.add8643.

7. Pacesa, M., Loeff, L., Querques, I., Muckenfuss, L.M., Sawicka, M., and Jinek, M. (2022). R-loop formation and conformational activation mechanisms of Cas9. Nature *609*, 191–196. https://doi.org/10.1038/s41586-022-05114-0.

8. Sun, W., Cheng, Z., Wang, J., Yang, J., Li, X., Wang, J., Chen, M., Yang, X., Sheng, G., Lou, J., and Wang, Y. (2023). AcrIIC4 inhibits type II-C Cas9 by preventing R-loop formation. Proc. Natl. Acad. Sci. USA *120*, e2303675120. https://doi.org/10.1073/pnas.2303675120.

9. Ouyang, J., Yadav, T., Zhang, J.-M., Yang, H., Rheinbay, E., Guo, H., Haber, D.A., Lan, L., and Zou, L. (2021). RNA transcripts stimulate homologous recombination by forming DR-loops. Nature *594*, 283–288. https://doi.org/10.1038/s41586-021-03538-8.

10. Jalan, M., Brambati, A., Shah, H., McDermott, N., Patel, J., Zhu, Y., Doymaz, A., Wu, J., Anderson, K.S., Gazzo, A., et al. (2025). RNA transcripts serve as a template for double-strand break repair in human cells. Nat. Commun. *16*, 4349. https://doi.org/10.1038/s41467-025-59510-x.

11. Ohle, C., Tesorero, R., Schermann, G., Dobrev, N., Sinning, I., and Fischer, T. (2016). Transient RNA-DNA Hybrids Are Required for Efficient Double-Strand Break Repair. Cell *167*, 1001–1013.e7. https://doi.org/10.1016/j.cell.2016.10.001.

12. Ortega, P., Mérida-Cerro, J.A., Rondón, A.G., Gómez-González, B., and Aguilera, A. (2021). DNA-RNA hybrids at DSBs interfere with repair by homologous recombination. eLife *10*, e69881. https://doi.org/10.7554/eLife.69881.

13. Platt, R.J., Chen, S., Zhou, Y., Yim, M.J., Swiech, L., Kempton, H.R., Dahlman, J.E., Parnas, O., Eisenhaure, T.M., Jovanovic, M., et al. (2014). CRISPR-Cas9 knockin mice for genome editing and cancer modeling. Cell *159*, 440–455. https://doi.org/10.1016/J.CELL.2014.09.014.

14. Kaltenbacher, T., Löprich, J., Maresch, R., Weber, J., Müller, S., Oellinger, R., Groß, N., Griger, J., de Andrade Krätzig, N., Avramopoulos, P., et al. (2022). CRISPR somatic genome engineering and cancer modeling in the mouse pancreas and liver. Nat. Protoc. *17*, 1142–1188. https://doi.org/10.1038/s41596-021-00677-0.

15. Chen, L., Chen, J.-Y., Zhang, X., Gu, Y., Xiao, R., Shao, C., Tang, P., Qian, H., Luo, D., Li, H., et al. (2017). R-ChIP Using Inactive RNase H Reveals Dynamic Coupling of R-loops with Transcriptional Pausing at Gene Promoters. Mol. Cell *68*, 745–757.e5. https://doi.org/10.1016/j.molcel.2017.10.008.

16. Fausto, N., Campbell, J.S., and Riehle, K.J. (2012). Liver regeneration. J. Hepatol. *57*, 692–694. https://doi.org/10.1016/j.jhep.2012.04.016.

17. Darlington, G.J., Bernhard, H.P., Miller, R.A., and Ruddle, F.H. (1980). Expression of liver phenotypes in cultured mouse hepatoma cells. J. Natl. Cancer Inst. *64*, 809–819.

18. Hashemolhosseini, S., Nagamine, Y., Morley, S.J., Desrivières, S., Mercep, L., and Ferrari, S. (1998). Rapamycin Inhibition of the G1 to S Transition Is Mediated by Effects on Cyclin D1 mRNA and Protein Stability. J. Biol. Chem. *273*, 14424–14429. https://doi.org/10.1074/jbc.273.23.14424.

19. Levesque, S., Kawashima, N., Hwang, G., Becerra, B., Schoonenberg, V.A.C., Mannherz, W., Homfeldt, L., Pinello, L., Agarwal, S., and Bauer, D.E. (2025). In cellulo DNA assembly for targeted genomic integration and rearrangement in human cells. Preprint at bioRxiv. https://doi.org/10.1101/2025.06.16.659926.

20. Nishiyama, J., Mikuni, T., and Yasuda, R. (2017). Virus-Mediated Genome Editing via Homology-Directed Repair in Mitotic and Postmitotic Cells in Mammalian Brain. Neuron *96*, 755–768.e5. https://doi.org/10.1016/j.neuron.2017.10.004.

21. Williams, D.A., Kohn, D.B., and Thrasher, A.J. (2025). Ex vivo modification of hematopoietic stem and progenitor cells for gene therapy. Mol. Ther. *33*, 2141–2153. https://doi.org/10.1016/j.ymthe.2025.03.058.

22. Wang, X., Liao, Y., Liu, D., Zheng, J., and Shi, M. (2025). Presetting CAR-T cells during *ex vivo* biomanufacturing. Mol. Ther. *33*, 1380–1406. https://doi.org/10.1016/j.ymthe.2025.02.031.

23. Llanos-Ardaiz, A., Bilbao-Arribas, M., de Galarreta, M.R., Lantero, A., Neri, L., Mauleon, I., Ferrer, V., Combal, J.P., Aldabe, R., and Gonzalez-Aseguinolaza, G. (2025). Genome Wide rAAV Integration Analysis in a Tyrosinemia Mouse Model Revealed Preference for R-loops in Actively Transcribed Genes and in the Absence of Homology Arms. Mol. Ther. *33*, 1–1667. ASGCT 28th Annual Meeting Abstracts. https://doi.org/10.1016/j.ymthe.2025.04.028.

24. Lin, S., Staahl, B.T., Alla, R.K., and Doudna, J.A. (2014). Enhanced homology-directed human genome engineering by controlled timing of CRISPR/Cas9 delivery. eLife *3*, e04766. https://doi.org/10.7554/eLife.04766.

25. Hirsch, M.L. (2015). Adeno-associated virus inverted terminal repeats stimulate gene editing. Gene Ther. *22*, 190–195. https://doi.org/10.1038/gt.2014.109.

26. Xu, Y., Jiao, Y., Liu, C., Miao, R., Liu, C., Wang, Y., Ma, C., and Liu, J. (2024). R-loop and diseases: the cell cycle matters. Mol. Cancer *23*, 84. https://doi.org/10.1186/s12943-024-02000-3.

27. Schiroli, G., Conti, A., Ferrari, S., Della Volpe, L., Jacob, A., Albano, L., Beretta, S., Calabria, A., Vavassori, V., Gasparini, P., et al. (2019). Precise Gene Editing Preserves Hematopoietic Stem Cell Function following Transient p53-Mediated DNA Damage Response. Cell Stem Cell *24*, 551–565.e8. https://doi.org/10.1016/j.stem.2019.02.019.

28. Pacesa, M., Pelea, O., and Jinek, M. (2024). Past, present, and future of CRISPR genome editing technologies. Cell *187*, 1076–1100. https://doi.org/10.1016/j.cell.2024.01.042.

29. Ran, F.A., Hsu, P.D., Wright, J., Agarwala, V., Scott, D.A., and Zhang, F. (2013). Genome engineering using the CRISPR-Cas9 system. Nat. Protoc. *8*, 2281–2308. https://doi.org/10.1038/nprot.2013.143.

30. Pavel-Dinu, M., Wiebking, V., Dejene, B.T., Srifa, W., Mantri, S., Nicolas, C.E., Lee, C., Bao, G., Kildebeck, E.J., Punjya, N., et al. (2019). Gene correction for SCID-X1 in long-term hematopoietic stem cells. Nat. Commun. *10*, 1634. https://doi.org/10.1038/s41467-019-09614-y.

OMTN, Volume *37*

Supplemental information

Potential effects of endogenous RNA/DNA hybrids on CRISPR-Cas9-mediated homology-directed repair

Francesco Puzzo, Batuhan Bayram, Claudia Macaubas, Angela Lin, Hagoon Jang, Feijie Zhang, Elizabeth Mellins, and Mark A. Kay

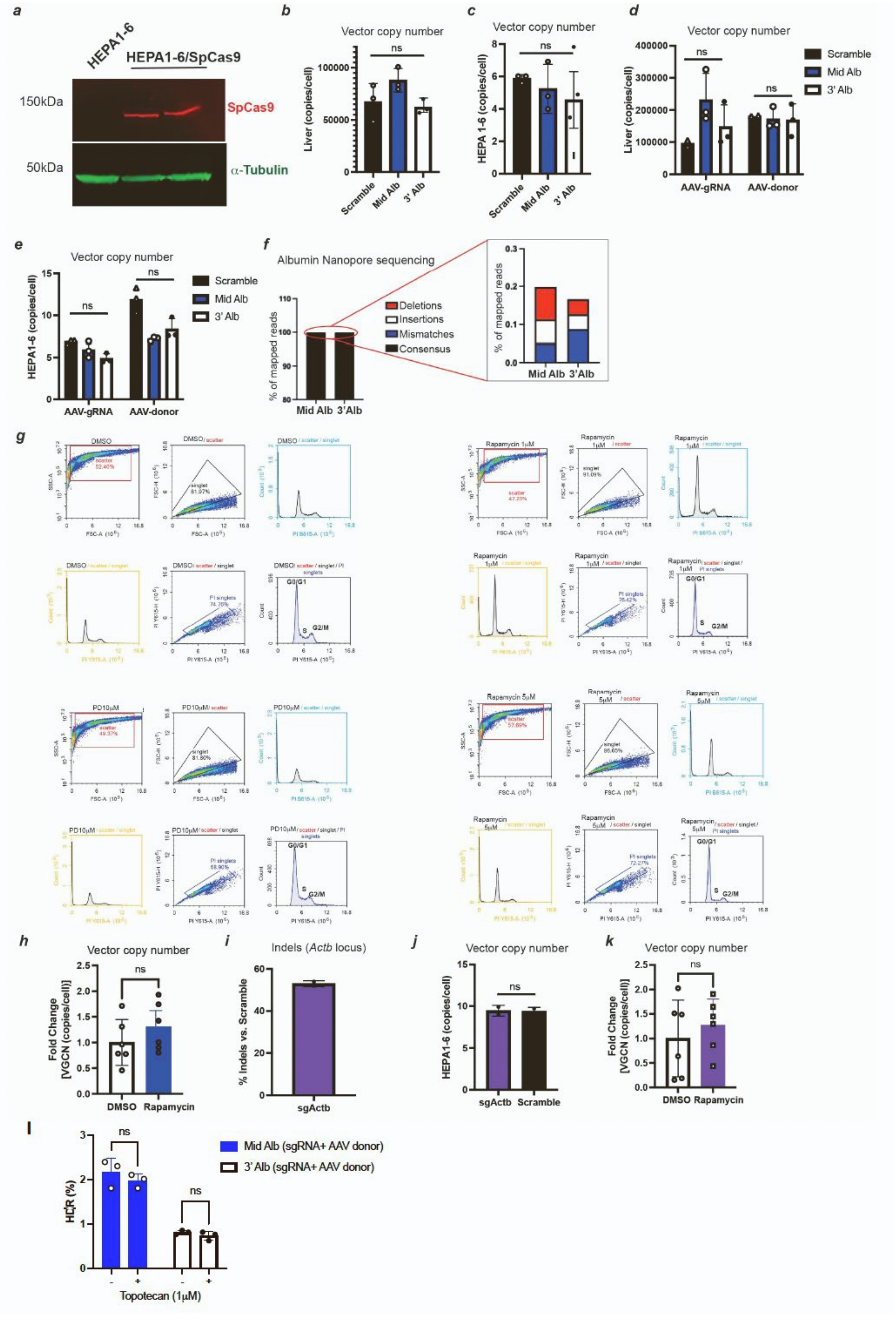
a
HEPA1-6
HEPA1-6/SpCas9
150kDa
50kDa
SpCas9
α-Tubulin
b
Vector copy number
Liver (copies/cell)
Scramble
Mid Alb
3' Alb
ns
c
Vector copy number
HEPA 1-6 (copies/cell)
d
Vector copy number
Liver (copies/cell)
AAV-gRNA
AAV-donor
Scramble
Mid Alb
3' Alb
e
Vector copy number
HEPA1-6 (copies/cell)
f
Albumin Nanopore sequencing
% of mapped reads
Deletions
Insertions
Mismatches
Consensus
Mid Alb
3'Alb
g
DMSO
PD10μM
Rapamycin 1μM
Rapamycin 5μM
G0/G1
S
G2/M
h
Vector copy number
Fold Change [VGCN (copies/cell)]
DMSO
Rapamycin
i
Indels (Actb locus)
% Indels vs. Scramble
sgActb
j
Vector copy number
HEPA1-6 (copies/cell)
sgActb
Scramble
k
Vector copy number
Fold Change [VGCN (copies/cell)]
DMSO
Rapamycin
l
HDR (%)
Mid Alb (sgRNA+ AAV donor)
3' Alb (sgRNA+ AAV donor)
Topotecan (1μM)

Figure S1. Analysis of AAV vector copy number and editing outcomes

a. Western blot analysis showing the expression of SpCas9 in HEPA1-6/SpCas9 cells. HEPA1-6 were used as negative control. Quantification of AAV vector copy number (VCN) in mouse liver (**b, d)** and HEPA1-6/SpCas9 cells (**c, e**) following administration of *scAAVDJ-sgMid Alb* or *scAAVDJ-sg3' Alb*, alone (**b, c**) or in combination with *ssAAVDJ-donor Mid Alb* and *ssAAVDJ-donor 3' Alb* (**d, e**). **f**. Nanopore analysis of integrants at the *Albumin* locus in HEPA1-6/SpCas9 cells transduced for 72 h with 10,000 vg/cell of *scAAVDJ-sgMid Alb* + 10,000 vg/cell of *ssAAVDJ-donor Mid Alb*, or 10,000 vg/cell of *scAAVDJ-sg3' Alb* + 10,000 vg/cell of *ssAAVDJ-donor 3' Alb.* **g.** Representative flow cytometry plots showing gating strategy for HEPA1-6 cells for cell cycle analysis using propidium iodide staining (PI). **h.** Fold change in HEPA1-6/SpCas9 cells VCN transduced with *scAAVDJ-sgMid Alb + ssAAVDJ-donor Mid Alb*, or *scAAVDJ-sg3' Alb + ssAAVDJ-donor 3' Alb*. upon treatement with rapamycin. DMSO-treated cells were used as control. **i.** Indel frequency at the *Actb* locus in HEPA1-6/SpCas9 cells transduced with *scAAVDJ-sgActb* compared to scramble control. **j.** AAV vector copy number in HEPA1-6/SpaCas9 cells transduced with *scAAVDJ-sgActb* or scramble control. **k.** Fold change in HEPA1-6/SpCas9 cells VCN transduced with 10,000vg/cell of s*scAAVDJ-sgActb + ssAAVDJ-donor Actb* upon treatment with rapamycin. DMSO-treated cells were used as control. **l.** ddPCR detection of HDR events in HEPA1-6/SpCas9 cells pre-treated with 1 µM topotecan for 1 h, followed by 72 h transduction with 10,000 vg/cell of scAAVDJ-sgMid Alb + 10,000 vg/cell ssAAVDJ-donor Mid Alb, or scAAVDJ-sg3′ Alb + ssAAVDJ-donor 3′ Alb.

Statistical analysis: **b- e.** One-way ANOVA with Dunnett's post hoc test; **h, j, k.** Student's t-test. *p*<0.05, **p*<0.01, ***p*<0.001, ****p*<0.0001, ns=non statistically significant. Error bars represent mean ± SD.

Table S1 (see file excel published along with supplemental material) contains:

- The raw data from the Nanopore sequencing experiment showed in figure S1f
- The sequences of the oligonucleotides used in the present study
